# Rheumatoid Inflammation of the Joints in Women

**Published:** 1878-07

**Authors:** 


Rheumatoid Inflammation of the Joints in Women.—It has occurred to me on several occasions to observe an affection of the joints, which, from the definite character of its symptoms and course seems to deserve a distinguishing name. From the fact that the condition of the articulation attacked resembles very much that of acute rheumatism, I think that the term "Rheumatoid" may aptly be applied to the disease. It should at the same time be remembered that I do not desire to imply that it is in any way connected with so-called chronic rheumatic arthritis.

Most of the cases I have seen have been admitted into the medical wards as suffering from some form of rheumatism. I cannot recollect having ever seen a joint disease in the male exhibit the symptoms which I am about to describe. In all the females, on the other hand, whom I have seen suffering from this affection, it has been associated with some uterine or vaginal irritation.

*Case* 1.—About ten years ago, when I was house-surgeon at Guy's Hospital, I was asked by one of the nurses to see her daughter, who had been recently married, and had got a bad elbow. The girl was about twenty years of age, and in the third or fourth month of pregnancy. Her right elbow was red, much swollen and acutely painful. She had had no injury to the joint, and I forget to what she attributed her trouble. I quite expected that it was about to suppurate, and was much surprised when after watching it carefully for two or three weeks, I saw the inflammatory symptoms gradually subside. Finally she got well, but was left with considerable stiffness of the elbow.

*Case* 2.—A woman, aged twenty-two, came to me as an out-patient in March, 1873, with the following history: She had been married three and a half weeks before. Three days afterwards she was at Woolwich Gardens, and was there exposed to cold. Soon after she noticed pains in her left hip, then "rheumatics" in her right arm, which had been bad ever since. The swelling, however, had only been considerable for about five days. There was redness and œdema of the whole of the right arm down to the fingers. It was especially large at and above the elbow, and in this situation it was very tender. I could feel no fluctuation, and I could not detect any cord-like induration along the course of the veins, in support of my first impression that she was suffering from phlebitis. Her general health was good. She was kept lying down with the arm swathed in cotton-wool, and in five or six weeks the swelling had gone, but the joint remained very stiff. Nine weeks after I first saw her she could only move the elbow through about ten degrees. I then used force to break down the adhesions, and I cannot say whether she ultimately recovered the full use of the arm, as she ceased attending. In this case I have no record as to pregnancy or leucorrhœal discharge.

*Case* 3.—In July, 1874, S. W., twenty-one years of age, was admitted into the hospital two months after her marriage, suffering from a swollen knee. Four or five weeks previously she had noticed pain in that joint; then her right shoulder had swelled. Finally the left knee, which had recovered, became so painful that she could not walk. Her ankle also had been swelled for a short time. On admission the right knee was two inches larger than the other, and very tender. The skin over it was reddened. She was unable to bend the joint, and passive movement was very limited. Her catamenia were regular. After five weeks' treatment, with rest and a splint, she went out, able to walk about fairly, but still rather lame from stiffness of the joint. While in the ward she had two or three attacks of severe abdominal pain, but I can find no facts to indicate whether this was of an ovarian origin, or from any other cause.

*Case* 4.—H. J., aged twenty-five, married four years, was admitted on 4th December, 1875, nearly a month after the birth of her second child. One week before her confinement the right knee had become swollen. She thought it was "the rheumatics." The right shoulder and carpus became also painful, and in the latter there was redness and swelling. She had to go to bed, and one week later she had an easy delivery, but suffered greatly at the time from the pain in the knee. When I saw her the knee was still red and swollen, and had become stiff in the bent position. I had to straighten it under chloroform, and she went out able to walk with a stiff but straight knee.

*Case* 5.—In March, 1877, I was asked by one of our physicians to see M. W., who was suffering from inflammation of the ankle-joint, which was thought to be of a strumous character, and likely to suppurate. She was twenty-two years of age, and had been married nine months. About three months before she had been an out-patient under the Obstetric Physician, suffering from some difficulty in retaing her urine as she walked about. It was then noticed that she was pregnant. A little later she again was attended with some vaginal discharge. Three and a half weeks before admission, pain came in her left hand, and three days after her left foot became swollen and painful.

She then took to her bed. When I saw her she was in the sixth month of pregnancy. Her left ankle was very tender, red and much swollen. Fluctuation could be felt about the inner malleolus. There had been a good deal of feverishness, but her temperature was then but little above normal. She was kept in bed and a conium poultice applied. In a week the redness went away. The ankle was now kept at rest by means of a splint. In five weeks the tenderness and fluctuation had disappeared, and she went out well, with the exception of stiffness of the joint, for which, about four months later, she was again for a short time under treatment without much benefit.

*Case* 6.—E. B., aged sixteen, unmarried, was admitted into a medical ward in May, 1877. She had been in service, and four days before her admission the attack had begun with slight shivering, followed by a pain in the right elbow. The shoulder of the same side and the other elbow were also for a short time affected. On admission there was slight feverish disturbance, but her temperature was never observed to be higher than 100 degrees. Twelve days later I saw her, and found the right elbow much swollen, hot, painful, flushed, but not fluctuating. There was also œdema to some distance from the joint. The condition of the joint led me to suspect a vaginal or uterine cause, and I made some inquiries, which elicited that she was in the fourth month of pregnancy. She was transferred to a surgical ward and remained under my care about six weeks. The redness and swelling slowly subsided, but the joint remained stiff. From time to time I broke down the adhesions, but she still found movement painful, and she had but little power in her elbow when I last saw her. I may add that she told us her father was rheumatic, and also that in the medical reporter's notes of her case I find that a systolic bruit was noticed at the base of the heart.

To these notes I might add a case of inflammation of the carpus, followed by stiff wrist, in a pregnant woman, and also two cases of inflammation of the same region in women in whom I could find no other cause than obstinate leucorrhœa. I might also include several cases of joint affection of a similar character following parturition. These, however, may be said to belong to the class of pyæmic inflammations, so I prefer to omit them, although I conceive that they should usually be placed in the same category with the cases which I have just narrated.

I would submit therefore that there is a definite joint-disease characterized by great pain and tenderness, and more especially by redness and œdema of the soft parts in the neighborhood. It is accompanied in the earlier stages by febrile disturbance, which, however, I have never noticed to be considerable. Usually a mild attack is noticed in other joints before the inflammation, so to speak, concentrates itself in one particular locality. The diseases with which it may be confounded are erysipelas, phlebitis, or acute suppuration of the joint. In the latter case the mistake might lead to serious consequences. The surgeon might be induced to make a free incision, and thus convert an inflammation which would have undergone resolution into a lingering suppuration. From most cases of erysipelas the absence of vesication and a high temperature would form sufficient means of discrimination. From phlebitis the absence of the cord-like induration of the veins would probably enable us to diagnose it, although the œdema in some of my cases resembled very much that which accompanies thrombosis. If I may form an opinion from the limited experience I have had, the prognosis is so far favorable in that the inflammation will probably go away without suppuration. The joint will, however, remain more or less bound by fibrous adhesions; so the patient must expect some impairment of the usefulness of the limb. As I have never had an opportunity of examining the condition of the joint after death or amputation, I cannot speak with confidence upon the pathological character of the affection. Judging from the absence of fluctuation in most of my cases and the presence of superficial œdema, I have been led to think that the chief seat of inflammation is in the fibrous capsule of the joint rather than in the synovial membrane. If the latter had been primarily affected I should have looked for effusion inside the articulation, and consequent fluctuation, as in the ordinary form of synovitis.

With respect to the causation of the disease, it will be observed that in all the cases reported or mentioned there was reason to suspect uterine or vaginal irritation. Some had severe leucorrhœa; others were pregnant, and of these all but one for the first time. In two cases there was no statement as to pregnancy, but as they had been married a short time previously, it is probable that pregnancy, vaginal discharge, or frequent sexual congress was the source of irritation.

I have said before that I have not seen such a disease in the male. The only exception I would make to this statement is in the case of carpal and tarsal disease. Here there is often much redness and œdema as well as acute pain, and such inflammations, without suppuration, are not infrequent in the gouty and rheumatic of both sexes. Moreover, I have never seen a case of joint affection like those I have narrated in the female, except where there was evidence of some vaginal or uterine irritation.

It may be alleged that these inflammations are merely examples of gonorrhœal synovitis in the famale, and I am disposed to admit that there is some alliance between the two affections in respect to their reflex origin through the nervous system. In the male, however, we rarely see any other joint affected than the knee, and the disease resembles synovitis. In the women whose cases I have narrated, the elbow was quite as often affected as the knee. There was, moreover, great œdema and redness, with little, if any, effusion into the joints The subsequent history was also unlike what is observed in gonorrhœal rheumatism. I cannot lay much stress upon the absence of a history of vaginal discharge in most of my cases, as probably no questions were asked upon that point, and even if there had been, the answers would not be reliable, unless an examination had been made.

If it is supposed that there was some vaginal discharge in each of the cases, which give rise to the joint inflammation, it can hardly have been an accidental coincidence that so large a proportion of them should have occurred in prgnant women. I cannot find that works upon midwifery make any mention of the liability during pregnancy to such affecticn. Nevertheless, the facts which I have brought forward seem to show that pregnancy either directly caused the inflammation by some influence reflected upon the vaso-motor system, or indirectly assisted in its development in a patient suffering from gonorrhœa or leucorrhœa, by the debility due to the pregnant condition.—*Obstetrical Journal.*

